# Autism Scientists’ Reflections on the Opportunities and Challenges of Public Engagement: A Qualitative Analysis

**DOI:** 10.1007/s10803-018-3783-7

**Published:** 2018-10-24

**Authors:** Gregory Hollin, Warren Pearce

**Affiliations:** 1 School of Sociology and Social Policy, University of Leeds, Leeds, LS2 9JT UK; 2 Present Address: iHuman, Department of Sociological Studies, University of Sheffield, Sheffield, S10 2TU UK

**Keywords:** Public engagement, Autism advocacy, Qualitative research, Ethics

## Abstract

This article draws upon qualitative interviews in order to examine how UK based research psychologists understand public engagement activities and interactions with autistic advocates. Researchers describe public engagement as difficult and understand these difficulties as stemming from autistic impairments. In particular, it is reported that a heterogeneity of autism impairments means there is little agreement on the form research should take, while socio-communicative impairments make interactions difficult. Conversely, researchers describe autistic individuals as having the capacity to positively influence research. In this paper we discuss the nature of these claims and stress the need for autism-specific modes of engagement to be developed.

## Introduction

In recent years there has been an increasing drive to meaningfully engage users, publics, and stakeholders with scientific research (Gibbons et al. 1994; Guston 2014). This drive for engagement is often imposed ‘top down’ by funding bodies such as the Economic and Social Research Council in the United Kingdom, the National Science Foundation in the United States, and the European Commission, all of which require a research ‘impact agenda’ to be written into grant proposals (Boswell and Smith 2017; Greenhalgh et al. 2016; Lok 2010). The drive is also ‘bottom up’, however, as increasing numbers of scientists recognize the important contributions that can be made by those outside of the academy (Watermeyer 2014; Wehrens 2014).

Public engagement is less of a novelty for autism scientists than it is for researchers in many other fields. Histories of autism in both the US (Eyal et al. 2010; Silverman 2012) and the UK (Evans 2017) demonstrate that since at least the 1960s psychiatrists and psychologists have engaged in extensive dialogue with parental advocates and activists who have radically shaped the nature of scientific research. Further, and at least since Jim Sinclair’s influential paper ‘don’t mourn for us’ (Sinclair 1993), researchers have also needed to consider the voices of autistic^1^ individuals themselves. Although it is argued that autistic voices have not been incorporated as fully as possible (Milton and Bracher 2013) and that there remains a degree of distrust on the part of autistic community (Milton 2014; Pellicano et al. 2014a), there is still significant evidence that autistic individuals have shaped autism science (Hart 2014). In particular, the emergence of a ‘neurodiversity movement’ which challenges the ‘tragedy’ or ‘medical’ model of autism is perceived to have been highly influential (Kapp et al. 2013).

In order that impact agendas may be best shaped to the needs of the autistic community, a small body of research into public engagement has begun to systematically consider the research deemed most important to autistic individuals. This research has determined that much of the autistic community believes that research oriented to the resolution of day-to-day difficulties should occupy a more central position than is currently the case (Pellicano et al. 2014b). This literature also notes that many autistic individuals have numerous ethical concerns with scientific research (Daley et al. 2013). As is the case in other areas (Shakespeare 1995; Thomas and Rothman 2016), many of these concerns orient around the possible eugenic potential of research leading to pre-natal diagnostics or autism cures. These are not perspectives held by all individuals, however, and fundamental disagreements within the autistic community mean that consensus on ‘acceptable’ research is unlikely and that various democratic mechanisms need to be forged in order to determine appropriate scientific responses to concerns raised (Russell et al. 2017).

While public engagement activities ensure that the views of autistic communities are becoming increasingly well known, a smaller body of research has also considered researchers’ views on engagement. Pellicano and colleagues (Pellicano et al. 2014a) found significant convergence between the views of autistic individuals, their parents, and researchers but also noted that researchers were less concerned about public service provision and autistic individuals’ ‘place in society’ than other stakeholders. Pellicano et al. also found divergent views within the scientific community on the importance and form that engagement should take. As similarly reported elsewhere (Carrington et al. 2016) it was found that researchers consider themselves to be more engaged with stakeholders than do autistic individuals or their families.

This existing research into public engagement provides important insights into the priorities of both researchers and autistic individuals. What has not been provided, however, is an in-depth consideration of the experiences, hopes, fears, and understandings of researchers *during* engagement activities. Given that engagement is necessarily dialogic and shaped by researchers’ perceptions and beliefs, we suggest that understanding researchers’ experiences is of crucial importance if future engagements are to be successful. In this paper we address this gap in the literature by drawing upon qualitative interviews in order to analyze researchers’ claims about the undertaking of public engagement activities.

Our core finding is that researchers describe engagement activities as particularly difficult due to an interaction between substantive political differences within the autistic community and impairments specific to autism. While existing research has noted conflict and political difference within the autism community (Pellicano and Stears 2011; Russell et al. 2017), that impairments—most notably socio-communicative impairments—are perceived by some of our interviewees to significantly affect engagement is an important and novel finding which has consequences for determining the nature of future engagement activities.

We draw upon the sociological and political science literatures in order to help us both understand and act upon these conclusions. Political scientist Pielke Jr. argues that divergent views and a multiplicity of perspectives, like those reported here, are reasonably common in discussions of scientific research (Pielke Jr. 2007). Pielke Jr. use the ideal-types of ‘tornado politics’ and ‘abortion politics’ in order to consider how value dissensus may affect the role of scientific knowledge in public deliberation and decision-making. A group of people in the path of a tornado can agree that their goal is to avoid said tornado and use scientific knowledge to help achieve their goal. In the case of abortion, however, value-laden matters such as morality, religion, and human rights may outweigh scientific knowledge in decision-making processes. Pielke Jr.’s argument, in sum, is that when there is significant value consensus scientific knowledge (‘tornado politics’) may straightforwardly guide community action. When there is disagreement and value dissensus (‘abortion politics’), however, it is unlikely that additional scientific knowledge will lead to political consensus.

Brown (2015) and Miller (2001) extend upon Pielke Jr’s work, arguing that when there is value dissensus and questions of power, morality, justice, and group identity are raised, science can become a site of politics. Here science becomes politicized, not in the pejorative sense of partisan interference with research, but as a “process whereby people persistently and effectively challenge established practices and institutions, thus transforming them into sites or objects of politics” (Brown 2015, p. 7). Brown argues that contestation and deliberation over political differences may be productive (Brown 2009), affecting both the direction of science and its constitutive practices (Hartley et al. 2017). These findings, we suggest, are directly relevant for public engagement in autism science given the value dissensus that we report upon and we conclude this article by considering the way in which they may be used to shape future engagement practices.

## Methods

The analyses within this paper rely upon data obtained through qualitative interview and were part of a larger sociological project examining the science of autism which was conducted by the first author. Participants were eligible for inclusion in the project if they held an academic post (above PhD level) within a British university and had self-declared interests in psychology, neuroscience, and autism. While a minority of participants also held clinical positions and were therefore involved in some form of medical provision, participants were interviewed as academic researchers and questions were not oriented towards clinical matters.

Twenty academics participated in the study. The sample consisted of seven Professors, two Readers, one Senior Lecturer, one Associate Professor, two Lecturers, and seven Postdoctoral Researchers. This sample represents a good cross-section of researchers working in the UK although the possibility of selection bias is impossible to avoid in a project such as this: an additional 12 researchers declined to take part in the study, while a further 12 did not respond to request for interview.

In addition to existing knowledge of the field, potential participants suitable for interviewing were sought through a variety of means; internet searches, through discussion with autism scientists at the authors’ own academic institution, and by asking interviewees for further contacts. All participants who agreed to take part in the research provided fully informed consent prior to the interview and agreed for quotations to be used in subsequent publication. Approval for the study was secured through the School of Sociology and Social Policy at The University of Nottingham’s internal ethics procedures.

Interviews lasted between 38 and 73 min, with a mean length of 55 min. Interviews covered a range of topics but were oriented to the following areas of enquiry; (i) how the interviewee came to be interested in autism, (ii) the nature of autism itself, (iii) the participant’s research, (iv) the impact of neuroscientific methodologies upon autism science, (v) the role of stakeholder and public engagement in the research process. It is the last of these topics which is the concern of the current paper.

During the interview participants were explicitly asked what they felt about the involvement of autistic advocates and charitable organizations in research endeavours. After this explicit invitation to dwell upon stakeholder engagement, discussion was largely participant led and there was significant flexibility in the direction of the interview. This is an approach designed to allow interviewees the space to raise topics which are most important to them (Bryman 2008). Although all scientists offered opinions on autistic advocacy, participants were not chosen because of their public engagement activities, nor were participants asked to state or defend their record of engagement.

For the duration of the interview conversation was audio-recorded and subsequently transcribed in full by the first author. During the transcription process text was anonymized and extracts are presented here simply with a note of the interviewee’s job title at the time of interview. Some extracts presented also include utterances made by the interviewer (the first author) and these sections are preceded by an ‘I:’. The interviewee’s utterances during these interactions are tagged with an acronym based upon their job title (e.g. PD for Post-doctoral researcher). Extracts have been cleaned for legibility with repetitions and hesitations removed.

Once produced, transcripts were interrogated for sections concerning autistic advocacy and public engagement. These sections were then coded by hand so that any reoccurring themes within the data could be noted and explored. This coding procedure followed a general inductive method (Thomas 2006) where, rather than testing specific hypotheses, the core meanings were allowed to arise from the data. This is a widely used approach within the social sciences, and builds on previous literature highlighting that:

qualitative research…has a great deal to offer in understanding the experiences and practices of individuals with ASD [autism spectrum disorder], families and practitioners; and in gaining knowledge in the areas of communication, behaviour, and social interaction (O’Reilly et al. 2016, p. 356).

Here we focus on autism researchers as public engagement practitioners, but similar methods could be employed to investigate the meanings attached to public engagement by other key stakeholders. The quotes selected and presented here were chosen to provide coherent illustrations of wider themes that emerged during the analysis.

To be clear, we do not suggest that through the use of interview data we are able to obtain unproblematic access to the thoughts of research scientists, far less procure accurate descriptions of actual engagements (Potter and Hepburn 2005). These are sensitive topics under discussion and, like anyone else, scientists will be aware of the social milieu within which their utterances are produced. However, and like others who have interviewed similar groups (e.g. Fitzgerald 2014), we do not find evidence to support the claim that interviewees were being *particularly* strategic in these conversations. Similarly, we would not wish to claim that the views of these researchers stand unproblematically for the views of the entire research community, although they do represent an illustrative cross-section within the UK. No matter how partial we take the accounts offered here to be, however, we believe they should provoke reflection on public engagement within autism science.

In the following analysis we detail three related themes which emerged within interviews. The first theme suggests that disagreements within the autistic community are caused, in part, by the heterogeneity of the autistic condition. The second theme suggests that conflicts within the community are exacerbated by socio-communicative impairments which make it difficult for autistic individuals with divergent views to understand one another. The final theme suggests that, despite these difficulties, researchers recognize traits in autistic individuals which make them particularly valuable research partners. While we are clear throughout that we do not treat the scientists’ assertions uncritically, we do stress their importance for future public engagement activities.

## Analysis

### A Short Story of Impasse

Before detailing three core themes arising from the interviews in detail, we prefigure our analysis with a ‘short story of impasse’—a story detailed by a number of participants and which brings into relief various facets of autism engagement. The story, retold in these extracts by a Professor (interview 7), involves a public engagement debate over the nature of autism research which results in an impasse. During the debate one individual stands up and says ‘I am a neurodiverse person and you must respect me’. At this point another autistic individual invariably rises and says ‘if I could throw a switch tomorrow and get rid of it I would’. An argument ensues and no compromise or agreement is reached. The Professor recounting this story describes looking on in a state of bewilderment. Back in the comfort of their office, they muse on this situation and ask ‘who am I to say one way or the other?’ or, in a more despairing tone, ‘Well, you know, where does that leave me as a scientist looking in from the outside?’

This story captures something about the ways in which researchers describe lay engagement in autism science. In particular, we suggest that within these interviews a number of scientists describe engagements with autistic individuals as being consistently and radically shaped by the autistic impairment itself. Two aspects of this shaping are made evident from the above story, and which map onto key themes outlined in greater detail below. First, engagement is shaped by the heterogeneity of the autistic experience. Individuals may experience autism very differently to one another and reach very different conclusions about what constitutes valuable research. These stark differences of opinion make determining an agreed-upon path forward almost impossible. Second, the socio-communicative impairments typical of autism are described by the tellers of The Story as make any form of rapprochement or compromise particularly difficult. This is made apparent in the above story by the fact the two arguing individuals are apparently unable compromise or engage in deliberative dialogue. What is missed from the story, but is perhaps more positive, is that what several scientists (Reader, interview 3; Professor, interview 7) described as an autistic ‘way of being’ can contribute a significant amount to autism research. The same conviction and singlemindedness which is deemed problematic during some forms of engagement is, at other times, understood as making autistic participants valuable research partners—natural employers of the scientific method.

In the rest of this analysis we expand upon and detail the diverse ways in which, according to practicing scientists, engagement activities are shaped by autistic impairments. We focus upon the themes introduced above: autism heterogeneity, socio-communicative impairment, and ‘a way of being’ consistent with the use of the scientific method.

### Heterogeneity

As is evident in the above story, during interview a number of scientists report that autistic individuals have fundamental value disagreements over autism and the nature of research; this is a finding that is supported by the existing literature (Pellicano and Stears 2011; Russell et al. 2017). Importantly, fundamental value disagreements over autism are framed throughout interview as being, in part, caused by the heterogeneous nature of the condition itself. Autism is a notoriously heterogenous condition (Happé et al. 2006) and ‘heterogeneity’ itself is a term used by scientists in a heterogenous manner (Hollin 2017). For current purposes, however, ‘heterogenous’ can be taken to mean that there are significant inter-personal differences between autistic individuals. This may be a matter of individuals occupying different positions on an autism spectrum; for example, two people may have qualitatively similar characteristics but the prominence of these characteristics may differ widely. (So, for example, mutism may be said to be continuous with a dislike of social chit-chat.) Alternatively, two people may have qualitatively distinct characteristics; one individual may have particular sensory sensitivities which do not affect a second individual in the slightest.

Throughout interviews, scientists argued that the heterogeneous nature of autism makes it harder to incorporate an autistic voice into research, or to generalize from one autistic voice to a broader community. When one autistic individual suggests that research into sensory sensitivities should be prioritized, scientists are aware that others would disagree for the simple reason that, for them, sensory sensitivity is not part of the autistic experience at all.

This problem is made clear in the below extract wherein the generalizability of ‘an autistic identity’ is discussed:

I think there is this very strong notion of an autistic identity that seems important. Not being neurotypical and then some of the things you hear around the way people describe sensory experiences. I still want to hear more about how common I think those are within the autistic experience. I’m happy to hear individuals tell me that that’s their experience, but I’m not going to take that as what autism is internally for those who have autism because these are just the voices that happen to be heard at the moment and particular people are just more demanding that their voice is heard. So I think the [scientific] community almost feels that they know what the voice of autism is saying but we haven’t been listening to it for very long so I think I’d probably give it a little bit longer before we really feel that we know what it’s saying.

Here the interviewee, a Professor (interview 17), questions the ‘strong notion’ that all individuals with autism claim an identity premised upon their condition—that is, that all individuals identify as neurodiverse or ‘non-neurotypical’—and struggle with sensory difficulties. While this Professor is happy to hear about these aspects of the ‘autistic experience’, there is a marked reluctant to assume that this is ‘what autism is internally’ for all those with the condition. The researcher, then, disputes any notion of a value-consensus among autistic individuals and is reluctant to shape their research around those whose ‘voices that happen to be heard at the moment’.

That wariness about generalizing from voices that ‘happen to be heard’ is made more acute because active self-advocates are assumed to disproportionately come from the ‘high-functioning’ end of the spectrum. The following two extracts make that disproportionality clear and also make the stakes evident.

I do worry about the kinds of groups who play this [neurodiversity] card so forcefully that the wider public forgets that there are people on the autism spectrum who are frankly profoundly disabled. I think that’s the problem. (Senior Lecturer, interview 2)

And:

I think these people [neurodiversity advocates] tend to be high functioning and when you see someone who’s low functioning, who’s sitting in a corner, can’t communicate with anyone and seems constantly in turmoil and is so frustrated, then I think it is, yes, it’s a disorder. (Research Fellow, interview 12)

Across these extracts there appears an agreement that, at the ‘higher functioning’ end of the autism spectrum, autism should be understood as a difference or a condition. For those at the other end of the spectrum, however, scientists describe individuals who are ‘profoundly disabled’ and ‘constantly in turmoil and so frustrated’. As we have seen in the story introduced earlier, even amongst ‘high functioning’ individuals there are reported disagreements about whether it would be desirable to ‘switch it off’. It is important to note that many autistic activists and scholars reject the notion of an autism spectrum with ‘high’ and ‘low’ functioning individuals (Yergeau 2018). It is equally clear from these extracts, however, that for some scientists the concept of a spectrum with ‘high functioning’ individuals is a powerful framing device when it comes to understand engagement activities. What we suggest here is that one of the ways—although certainly not the only way—in which scientists explain differences between autistic individuals is through recourse to the heterogeneity of the autistic impairment itself; where someone sits on the spectrum, the particular traits they exhibit, and so forth.

The problems encountered by autism scientists appear typical of ‘abortion politics’ we discussed during the introduction (Pielke Jr. 2007). Accordingly, contending with significant value dissensus in relation to scientific research is hardly unique a unique problem. Indeed, scientists recognized this and, reflecting upon dissent and diversity with various communities (including the scientific community), compared this diversity to disagreements over autism. Similarly, another scientist noted their ‘general scepticism’ towards individuals who claim to speak for whole communities, regardless of whether those people are autistic self-advocates or ‘community leaders in Palestine’ (Professor, interview 7). What we suggest here is that autistic heterogeneity is taken by scientists to *exacerbate* this problem, although it is hardly unique to autism.

### Generating Sociability

In the previous section we argued that, during interview, scientists claim a lack of value consensus amongst the autistic community over the direction autism research should take and, further, that this dissensus was exacerbated by the heterogeneity of autistic experience. An additional barrier to the production of successful engagements, we suggest, is a reported difficulty in generating sociable relations between researchers and advocates.

In a comprehensive study examining the nature of successful engagement within genetics research, Panofsky argues that, quite aside from economics or control of resources, a key factor in successful researcher-advocate partnerships is the generation of personal relations (Panofsky 2011). ‘Generating sociability’, Panofsky contests, is core to both the initial forging, and subsequent shaping, of research partnerships. Singh (2016) and Silverman (2008) attest that such sociable relationships are found between researchers and autism parental advocacy organizations, while histories written by Silberman (2015) and Feinstein (2010) are replete with stories of highly charismatic parental advocates who are able to attract research interest and maintain influence. While there are huge numbers of charismatic individuals with autism (and researchers do describe themselves as particularly drawn to autistic individuals (Hollin and Giraud 2017)) Panofsky’s focus on sociability does pose questions of engagement in autism science.

A core trait associated with autism is socio-communicative impairment (American Psychiatric Association 2013). Two aspects of this socio-communicative impairment are relevant for the current discussion. First, many individuals may be uncomfortable in situations which require sustained interpersonal interaction. Second—and amongst UK-based psychologists this is likely to be a prominent area of academic interest given the history of research into Theory of Mind (Baron-Cohen et al. 1985)—autistic individuals may struggle to take another’s perspective.

It is important to note here that many authors who favour the Social Model of Disability and Neurodiversity movements reject this framing of autism (Yergeau 2018). Damien Milton, for example, describes socio-communicative breakdown as a matter of “reciprocity and mutuality” (p. 884) wherein two parties occupying very different lifeworlds *both* struggle to comprehend each other; no one is less able or more deficient and the breakdown is, instead, socially situated. While significant differences arise from these perspectives, and while we believe there are important gains to be made by following Milton’s lead, in the present context what is important is that, in both accounts, difficulties in communication between autistic individuals and scientists may be expected and several interviewees explicitly argued that impaired socio-communicative functioning was at least partially responsible for problematic engagements. Again, regardless of whether we agree or disagree with these researchers, these experiences and opinions are surely important in shaping engagement activities.

In the below extract a scientist recounts an experience wherein they received criticism for suggesting that ‘specialist interests’ weren’t ‘always a great thing’:

I mean I got in trouble with a lot of autism advocates for suggesting that having specialist interests wasn’t always a great thing. You know, they came down on me like a ton of bricks but, you know, then this is it, they can’t sometimes see that it’s not. (Reader, interview 1)

The conclusion reached by the interviewee here is worth considering. Following criticism, the scientist does not reconsider their original position that specialist interests can be problematic but nor do they suggest that the autistic individuals to whom they were speaking were incorrect; autistic heterogeneity ensures that different experiences of the matter in hand are possible, probable in fact. What the interviewee finds problematic in this interaction however is that ‘they can’t sometimes see that it’s not’; the inability to take another’s perspective ensures that they ‘come down’ on the scientist ‘like a ton of bricks’. It is the inability to recognize the diversity of the autistic experience which make engagement problematic.

A very similar point is made in the following extract, again concerned with sensory sensitivity:

One thing I have noticed with autistic people I’ve worked with is that they sometimes think that because they can’t bear light, they find it really difficult to grasp that not all autistic people will think like [that], you know, they have problems generalizing sometimes. (Professor, interview 20)

In contrast to disagreements about heterogeneity, in this extract the Professor specifically details something about autistic people as a particular group. ‘They find it really difficult to grasp’ the heterogeneity of the autistic experience, they ‘have problems generalizing’. This has the potential to create conflict between autistic individuals but also between advocates and scientific researchers.

The above point is discussed at length and with clear reference to autistic impairment, in the following extract:

I think one of the challenges is that one of the kind of key characteristics of autism is lack of insight and lack of ability to take somebody else’s point of view and I think this makes it really challenging. I mean I have been to meetings where there have been just massive rows and disagreements because one person with autism is saying one thing and somebody else affected by autism is saying something completely different and they just cannot accept that the other point is valid or should be heard, you know? So, I think it would be a big challenge to have some kind of group where all views could be expressed and taken into account. I mean you just have extremes, some people really thinking that you shouldn’t do intervention because we shouldn’t be able to cure autism because autism is a brilliant thing, other people saying “well of course we should [cure autism] because autism is terrible and we need to sort it out”. That’s quite difficult. (Reader, interview 1)

Once again, this scientist elucidates their point through reference to a public discussion wherein two autistic individuals engage in a lengthy row. It is explicitly stated here that this row occurs because of an *interaction* between divergent views and a ‘lack of insight and a lack of ability to take somebody else’s point of view…they just cannot accept that another point is valid or should be heard’. The end result is a researcher describing themselves as being in a particularly challenging, and confrontational environment. As a Professor states:

I suppose the answer is yes of course we take into account what they have to say because sometimes they have very interesting things to say. But on the other hand they have things which are just bewilderingly contradictory and so how do you take that on board? (Professor, interview 7)

The contributions of autistic individuals are ‘just bewildering contradictory’ and this, combined with an inability to compromise or take another’s perspective, makes it hard for a researcher to know what to do.

### An Incredible Capacity

The previous sections make it seem as though the scientists interviewed showed little willingness for engagement with autistic advocates. Such an image is not accurate, however, for scientists consistently said that they valued the input of autistic individuals and advocates.

Interviewees were positive about the ways in which engagement with autistic individuals had shaped their research, repeatedly claimed that they valued insight into the ‘inner experience’ of autism, and paid greater attention to the social and ethical consequences of their work on the basis of input from autistic individuals. One Reader argued that they were more attuned to the implications of their research following dialogue—‘The people I’ve worked with have most certainly shaped my social or my ethical stance on autism research’ (Reader, interview 3)—and this belief that ‘you learn so much from speaking to people with autism or families in terms of what’s important, what matters’ (Lecturer, interview 11) was widespread.

Researchers were also keen to stress that engagement with autistic individuals not only shaped research priorities but also shaped particular experiments or theoretical positions. This is made particularly clear in the following extract:

I: Can speaking to people with autism affect the research process?

L: Yeah, definitely. I mean it directly affects it by changing your experiments to suit them so they can do the experiments but also what they say makes you think and you try and put what they’re saying into your theory. (Lecturer, interview 15)

Indeed, the benefits of engagement can be so significant that some interviewees attempted to formalize relations and incorporate autistic voices into the laboratory group. A Professor makes exactly this point, saying of one individual:

We’re hoping to get them on our steering group in the lab’ because they’re genuinely insightful and thoughtful [and have] things to say about the inner experience of what it’s like for one person to have autism. (Professor, interview 7)

What is particularly noteworthy is that many of the same traits which were described as making engagement difficult are here articulated as particularly beneficial. The following interview extract speaks directly to the benefits of autistic experience for laboratory research. Here a Professor describes an autistic individual who is a practicing scientist and laboratory member:

They’re an amazing researcher. Our last paper they’re on that and I mean the contribution I mean they are just the most incredibly analytical thinker. I mean an absolute mine of information. You know, the way that people with autism won’t compromise with the truth and won’t cut corners and won’t do any of those things, you really see the advantages when you actually co-author a paper with somebody with autism. They’ve got a unique view of it but also just has an incredible analytical capacity. (Professor, interview 20)

Within this extract some of the same traits which were elsewhere described as problematic—for instance, that the individual in question ‘won’t compromise’—are here viewed positively. Within the laboratory ‘you really see the advantages’ of an autistic individual with a ‘unique view’ and ‘an incredible analytic capacity’. We see in this description the flip-side of the problematics presented earlier and there is a real sense that you can’t have one without the other.

## Conclusion

Drawing upon extensive interviews with practicing research scientists we have argued that many scientists understand engagement with autistic individuals as being shaped by an interaction of two factors. First, scientists argue that engagement with autism science is akin to ‘abortion politics’ (Pielke Jr. 2007) because there is widespread disagreement within the autistic community over the best course of action for scientific research to take. On this point our findings match those made in the existing literature (Pellicano and Stears 2011; Russell et al. 2017). Second, interviewees attributed the cause of these profound disagreements to the *autistic impairment itself*—first, because autism is such a heterogeneous condition and, second, because individuals with autism are understood as struggling to take others’ perspectives and as frequently being uncompromising. The other side of this particular coin, we have suggested, is that the very same skill set possessed by those with autism might actually make them particularly suitable research partners.

As noted previously, these data should not be treated uncritically; unlike the data used by many conversation analysts (O’Reilly et al. 2016), interview data are not naturally occurring and the words spoken in the context of a research interview should not be assumed to map on straightforwardly to interviewees’ thoughts. Nor should scientists’ descriptions of engagement events be assumed unproblematically depict what happens. Nor should those scientists who agreed to be interviewed be assumed to be entirely representative of the entire scientific community. In line with these reservations, we think it is important not take scientists’ utterances uncritically. The assertion that the autistic individual is the ‘problem’ in engagement, in particular, can and probably should be problematized (Milton 2012, 2014). Nonetheless, one does not need to be uncritical in order to take the experiences of these scientists seriously. What is described is a situation wherein autistic voices are not heard when it would be both ethical and scientifically productive for them to be so and, accordingly, condition specific mechanisms need to be put in place which allow a plurality of voices to be heard. To this end, we conclude with suggestions for improved engagements as well as noting areas in need of further study.

First, and in common with other areas of politicized science, it is important that dissensus is recognized and embraced (Rescher 1995; Sarewitz 2011). Pielke Jr. suggests that treating examples of ‘abortion politics’ *as if* they adhered to the principles of ‘tornado politics’—that is, *as if* there were value consensus about the ideal contribution of scientific research—is unlikely to be a successful strategy. Pielke Jr. suggests such attempts are likely to fail for as “long as there is dispute over values in a particular context, appeals to science can offer little to resolve those value differences” (Pielke Jr. 2007). In other words, increasing certainty about, e.g., the role of pre-natal testosterone in autism is unlikely to change anyone’s values. It is important that the scientific community recognize both the limits of their possible contribution in this regard and the views of divergent parties.

Second, we suggest that far greater effort needs to be invested in developing mechanisms and venues which allow fruitful dialogue. Brown argues that deliberative reasoning over conflict should be at the centre of bioethical debate (Brown 2009). The stories told by participants in this study strongly suggest that while Town Hall style debates are potentially good venues for exposing conflict they are likely very poor venues for deliberation. Accordingly, we think there are grounds to query their utility in autism engagement.

As an alternative to Town Hall debates, Pellicano and colleagues used focus groups as part of a mixed-methods study asking stakeholders what autism research should focus upon (Pellicano et al. 2014b). The focus groups in this study were “kept exclusive (e.g. researchers only, autistic adults only) to avoid potential disagreement between groups, p. 759” (Pellicano et al. 2014b). These focus groups improved significantly upon the aforementioned town hall debates by allowing a greater plurality of voices to be heard. Nonetheless, the exclusivity of these focus groups also entailed the curtailing of deliberation by keeping diverse perspectives apart. While the focus on plurality in this work is highly positive, following Brown (2009) we suggest that in a considered and well moderated environment, deliberation and conflict can be channelled productively. To take an example from another area of politicized science, climate change, Porter et al. demonstrate how a carefully moderated climate change blog acted as a locus for attempts to resolve tensions between polarized actors (Porter et al. 2018). It should not be assumed that such deliberative efforts can—or should—achieve consensus. However, they can be valuable as a means of navigating the political tensions within autism science in a more productive manner than the impasse of The Story related by some interviewees.

It seems highly likely to us that, as in this example from climate science, alternative modes of engagement will often orient around online activities, although the form these may take requires further investigation. It is well known that there are extensive and diverse autistic communities online (Bagatell 2007, 2010; Brownlow et al. 2006). Certain voices remain excluded from online spaces but it seems likely that a wide array of stakeholders may be able to take part in discussion and that online spaces may open avenues for increased deliberation and conversation. As Russell and colleagues demonstrate (Russell et al. 2017) there remains a need for researcher commitment to ensure that diverse and dissenting views are heard and future research should be undertaken to find mechanisms which ensure such practices.

## Footnotes

1 The term ‘autistic’ is used throughout the paper as research has suggested that the term is preferred by a large proportion of autistic adults (Kenny et al. 2015). We also draw a distinction between ‘impairment’ and ‘disability’ and avoid the term ‘deficit’ entirely. In line with the Social Model of Disability (Oliver 1990) we use ‘impairment’ to refer to an underlying cognitive or biological state. The term ‘disability’, meanwhile, should not be understood as ‘a corporeal [or cognitive] deficit but in terms of the ways in which social structure excludes and oppresses disabled people’ (Hughes 2002). This is a position largely consistent with ‘neurodiversity’ perspectives on autism, although we do not have space to explore the connections here (Kapp et al. 2013; Moore 2014; Yergeau 2018).
